# Antibiotic Resistance and Characteristics of *Vibrio parahaemolyticus* Isolated from Seafood Distributed in South Korea from 2021 to 2022

**DOI:** 10.3390/microorganisms13071566

**Published:** 2025-07-03

**Authors:** Jonghoon Lee, Hansol Kim, Haiseong Kang, Yongchjun Park, Insun Joo, Hyochin Kim

**Affiliations:** 1 Food Microbiology Division, Food Safety Evaluation Department, National Institute of Food and Drug Safety Evaluation, Cheongju 28159, Republic of Korea; whdgns46@korea.kr (J.L.); hskmfds@korea.kr (H.K.); rkdgotjd12@korea.kr (H.K.); yongchjun@korea.kr (Y.P.); jis901@korea.kr (I.J.); 2 College of Veterinary Medicine, Chungbuk National University, Cheongju 28644, Republic of Korea

**Keywords:** *Vibrio parahaemolyticus*, antimicrobial resistance, ampicillin, seafood, gastroenteritis

## Abstract

This study aimed to investigate the prevalence, antimicrobial resistance (AMR), and virulence characteristics of *Vibrio parahaemolyticus* (*V. parahaemolyticus*) isolated from olive flounder (*Paralichthys olivaceus*) and rockfish (*Sebastes schlegelii*) sashimi samples sold in South Korea from 2021 to 2022. A total of 500 fish samples were analyzed, from which 17 *V. parahaemolyticus* isolates were obtained. Antibiotic susceptibility testing using the minimum inhibitory concentration method revealed that 58.8% (10/17) of the isolates exhibited resistance to ampicillin, indicating the potential for AMR transmission in seafood-associated pathogens. Whole-genome sequencing (WGS) and a polymerase chain reaction detected the presence of *tlh* and *trh* virulence genes in all isolates, suggesting their pathogenic potential. Although the overall isolation rate of *V. parahaemolyticus* was low, the presence of virulence and AMR genes indicates public health relevance associated with raw seafood consumption. The increasing consumer demand for raw fish, coupled with environmental changes such as rising ocean temperatures, underscores the necessity of continuous surveillance to prevent foodborne outbreaks. These findings emphasize the need for targeted AMR monitoring and further research to mitigate the dissemination of resistant *V. parahaemolyticus* strains and enhance seafood safety.

## 1. Introduction

*Vibrio parahaemolyticus* is a Gram-negative, halophilic, zoonotic pathogen commonly found in marine environments, including aquatic organisms. Infections caused by *V. parahaemolyticus* are primarily associated with the consumption of raw or undercooked seafood, leading to gastrointestinal symptoms such as abdominal pain, nausea, vomiting, fever, and diarrhea [1]. However, severe cases can result in life-threatening foodborne illness, including sepsis and skin lesions, particularly in immunocompromised individuals.

The occurrence of *V. parahaemolyticus* infections is strongly influenced by environmental factors, particularly ocean temperature. As global warming accelerates the rise in sea temperatures, its prevalence as a foodborne microorganism is expected to increase, presenting a growing public health concern [2]. Additionally, climate change has been linked to genetic mutations in virulence factors, such as thermolabile hemolysin (*tlh*) and thermostable direct hemolysin (*tdh*), which may enhance the pathogen’s virulence [3,4].

South Korea, bordered by the ocean on three sides, has traditionally relied on seafood as a dietary staple. In recent years, growing consumer interest in health and nutrition has further boosted seafood consumption and aquaculture expansion [5]. However, large-scale aquaculture practices, while meeting consumer demand, have introduced various pathogens into farmed species, leading to increased antibiotic use. In particular, food- and waterborne pathogens such as *Vibrio parahaemolyticus* have been frequently detected in restaurant fish tanks in Seoul (50.7% prevalence), with isolates showing ampicillin resistance rates of over 50%, raising additional public health concerns about the emergence of antibiotic-resistant bacteria [6,7].

Among seafood products, olive flounder (*Paralichthys olivaceus*) and rockfish (*Sebastes schlegelii*) are among the most popular seafood choices in Korea, and are commonly consumed raw [8]. Advancements in aquaculture and cold-chain technology have improved the accessibility and availability of these fish in retail markets. However, their popularity as raw seafood increases the risk of exposure to various food- and waterborne pathogens, including *Salmonella* spp., *Norovirus*, and *Escherichia coli*, while *V. parahaemolyticus* in particular should also be considered due to its frequent association with seafood consumption [8].

*V. parahaemolyticus* develops antibiotic resistance through various mechanisms, including the production of β-lactamase enzymes, target site modifications, reduced antibiotic uptake, and efflux pump activation [9]. This study aimed to investigate the prevalence, antimicrobial resistance, and genetic characteristics of *V. parahaemolyticus* isolated from olive flounder and rockfish sashimi in South Korea from 2021 to 2022. Minimum inhibitory concentration (MIC) testing was performed according to Clinical and Laboratory Standards Institute (CLSI) guidelines. Whole-genome sequencing (WGS) and multilocus sequence typing (MLST) were conducted to identify genetic sequences and characterize antibiotic resistance genotypes [10].

Additionally, the presence of virulence factors *tlh* and *tdh* were detected using the Virulence Factor Database (VFDB). The genetic relationships of *V. parahaemolyticus* strains were analyzed to characterize their distribution in South Korea [11].

## 2. Materials and Methods

### 2.1. Sample Procurement

For this study, South Korea was categorized into six regions: Jeolla, Gangwon, Chungcheong, Seoul–Gyeonggi, and Gyeongsang. Although Seoul and Gyeonggi were treated as a single region for classification, samples were collected from both areas. A total of 500 seafood samples were collected over 2 years, with 250 samples obtained in 2021 and an additional 250 in 2022. In 2021, samples were distributed as follows: Jeolla (40), Gangwon (6), Chungcheong (63), Seoul–Gyeonggi (71), and Gyeongsang (70). In 2022, samples were collected as follows: Jeolla (40), Gangwon (10), Chungcheong (50), Seoul–Gyeonggi (80), and Gyeongsang (70) (Table 1 summarizes the sample distribution).

To prevent microbial growth, all samples were transported in iceboxes [12]. Upon arrival, they were immediately refrigerated until analysis to preserve their integrity. Laboratory experiments, including bacterial isolation and antibiotic susceptibility testing, commenced within 24 h of collection to prevent microbial alterations.

### 2.2. Enrichment and Isolation

The isolation of *Vibrio* species was conducted according to the Food Code established by the Ministry of Food and Drug Safety [13]. Each sample (25 g) was aseptically weighed and mixed with 225 mL of alkaline peptone water at a 9:1 diluent-to-sample ratio [11]. Samples were homogenized using a BagMixer (Interscience, Saint-Nom-la-Bretèche, France) and incubated at 35–37 °C for 24 h to facilitate bacterial enrichment.

After incubation, the enriched sample was streaked onto thiosulfate citrate bile salt sucrose (TCBS) agar and incubated at 37 °C for 18–24 h [14]. Five colonies with typical morphological characteristics of *V. parahaemolyticus* were selected and transferred onto tryptic soy agar plates supplemented with 2% NaCl. These plates were incubated at 35–37 °C for 18–24 h to promote colony growth. Pure colonies were then isolated and identified by matrix-assisted laser desorption/ionization time-of-flight mass spectrometry (MALDI–TOF MS) using the VITEK MS system (BioMérieux, Marcy-l’Étoile, France).

### 2.3. Antibiotic Susceptibility Testing

Antibiotic susceptibility testing was conducted via the minimum inhibitory concentration (MIC) method in accordance with the Clinical and Laboratory Standards Institute (CLSI) guidelines [15]. Test strains were cultured 1 day before the experiment, and a sufficient number of colonies were selected with a sterile loop and needle. The selected colonies were then suspended in 5 mL of 0.65% saline solution. The bacterial suspension was standardized to a 0.5 McFarland standard using a DensiCHEK Plus (bioMérieux, France) to ensure uniform turbidity.

Next, 10 µL of the standardized bacterial suspension was introduced into 11 mL of cation-adjusted Mueller–Hinton broth with TES (Thermo Scientific, T3462, Lenexa, KS, USA). The inoculated medium was thoroughly mixed, and 50 µL of the suspension was dispensed into each well of a 96-well microplate. The antimicrobial agents tested, their concentration ranges, and interpretive criteria for MIC determination are summarized in Table 2. The MIC breakpoints for streptomycin (STR), colistin (COL), and nalidixic acid (NAL) were designated as ‘ND’ (not determined), as CLSI has yet to establish breakpoints for *V. parahaemolyticus* [15].

### 2.4. PCR Detection of Toxins and Related Genes

Polymerase chain reaction (PCR) was performed to detect toxins and related genes. Key toxin genes of *V. parahaemolyticus*, including *tlh*, *tdh*, and *trh*, were analyzed. Additionally, secretion-related genes were examined [16,17,18]. The primer sequences used for PCR are listed in Table 3.

The *tlh* gene, which encodes a thermolabile hemolysin, was used for species-specific identification [16,18]. *tdh* and *trh*, which are virulence genes of *V. parahaemolyticus*, were detected via multiplex PCR [15]. Additionally, genes related to secretion systems (T3SS1, T3SS2α, T3SS2β, T6SS1, and T6SS2) were screened to evaluate their roles in bacterial pathogenicity [17,18,19,20].

### 2.5. WGS

WGS was conducted on antibiotic-resistant *V. parahaemolyticus* strains identified through MIC testing. Specifically, WGS was performed on 10 isolates that were resistant to ampicillin (AMP).

Genomic DNA (gDNA) was isolated with the MagListo™ 5M Genomic DNA Extraction Kit (Bioneer, Cat# K-3603, Daejeon, Republic of Korea). Extracted gDNA was quantified with a Qubit fluorometer (Qubit 3.0 or Qubit 4; Thermo Fisher Scientific, Cat# Q33240, Waltham, MA, USA) and qualitatively assessed with a NanoDrop 2000 UV-visible spectrophotometer (Thermo Fisher Scientific, Waltham, MA, USA).

Extracted gDNA was fragmented and processed for library construction using the Illumina DNA Prep Kit or Nextera DNA Flex Library Prep Kit (Illumina, Cat# 20018704, San Diego, CA, USA), and indexed with the Nextera DNA CD Index Kit (Illumina).

WGS was conducted on an Illumina MiSeq platform with the MiSeq Reagent Kit v3 (Illumina, Cat# MS-102-2003, San Diego, CA, USA). The generated FASTQ data were processed using CLC Genomics Workbench 12 (QIAGEN, Hilden, Germany) by trimming low-quality reads and performing genome assembly.

### 2.6. Gene Analysis and MLST

Antibiotic resistance genes were identified using the ResFinder tool (ver 4.5.0), hosted by the Center for Genomic (CGE, Technical University of Denmark, Lyngby, Denmark; https://www.genomicepidemiology.org/ (accessed on 28 June 2025)). Resistance genes were identified based on a 90% identity and 60% coverage threshold, following CGE guidelines.

MLST was performed using the MLST software (ver 2.0.9) to assign sequence types to the isolates [21]. The allelic profiles of *V. parahaemolyticus* were matched against the MLST database to assign sequence types (STs).

A phylogenetic tree based on MLST data was constructed using the MEGA X software (ver 10.2.6; Arizona State University, Tempe, AZ, USA) to assess genetic relationships among isolates using the neighbor-joining method with 1000 bootstrap replicates for statistical robustness [22].

### 2.7. Virulence Factor Analysis Using WGS Data

Virulence factors in *V. parahaemolyticus* strains were identified using VFanalyzer, a bioinformatics tool hosted by the Virulence Factor Database (VFDB, https://www.mgc.ac.cn/VFs/ (accessed on 28 June 2025)) [11]. The VFDB provides a curated database of known bacterial virulence genes, enabling the identification and classification of virulence-associated factors.

Identified virulence factors were categorized into two groups: common virulence factors and strain-specific factors. The classification of virulence genes was based on a comparison with core and accessory virulence factor models curated in VFDB.

## 3. Results

### 3.1. Isolation of V. parahaemolyticus from Fish Samples

Among 500 samples, 17 *V. parahaemolyticus* isolates were obtained. In 2021, the isolation rate was 3.2% (eight isolates out of 250 samples), while in 2022, it was 3.6% (nine isolates out of 250 samples) [Table 4]. Overall, the isolation rate was 3.4% (17 isolates out of 500 samples). All isolates were obtained from domestically produced seafood.

### 3.2. Antibiotic Susceptibility Test

All 17 isolates were tested for antibiotic susceptibility, with 58.8% (10/17) showing resistance to ampicillin. However, no resistance to other antibiotics, including tetracycline, was detected (Table 5) [15].

For nalidixic acid, all isolates had susceptibility values of ≥2. For streptomycin, only one isolate had a value of ≥32, while the remaining isolates had values of ≥16. For colistin, 11 isolates had values of ≥32; however, resistance could not be determined conclusively owing to the lack of established breakpoints.

### 3.3. PCR Detection of Toxin and Related Genes

All isolated strains were tested by PCR for the detection of virulence factors *tlh*, *tdh*, and *trh*. PCR results showed that *tlh* was detected in all strains, whereas *tdh* was absent in all strains (Table 6). Additionally, *trh* was detected in 12 of 17 strains.

T3SS1, associated with *tlh*, was detected in all strains, whereas T3SS2α and T3SS2β, associated with *tdh*, were not detected in any strain. T6SS1, which plays a role in bacterial competition and immune evasion, was found in 9 of 17 strains. Similarly, T6SS2, known to facilitate host interactions in aquatic environments, was detected in all strains.

### 3.4. WGS and MLST

The results from ResFinder and MLST analysis performed using the CGE server are summarized in Table 7.

Genotypic analysis revealed that all strains carried β-lactam resistance genes, including blaCARB variants. *blaCARB-40*, *blaCARB-21*, and *blaCARB-26* were the most prevalent variants. The phenotypic results from the MIC assays confirmed resistance to ampicillin. WGS analysis indicated that all strains carried resistance genes to penicillin-class antibiotics.

MLST analysis assigned isolates to multiple STs, with some isolates having novel or unassigned STs. Notably, ST114 was identified in two isolates, whereas several isolates were closely related to ST992, ST396, and ST3223.

**Figure 1:**
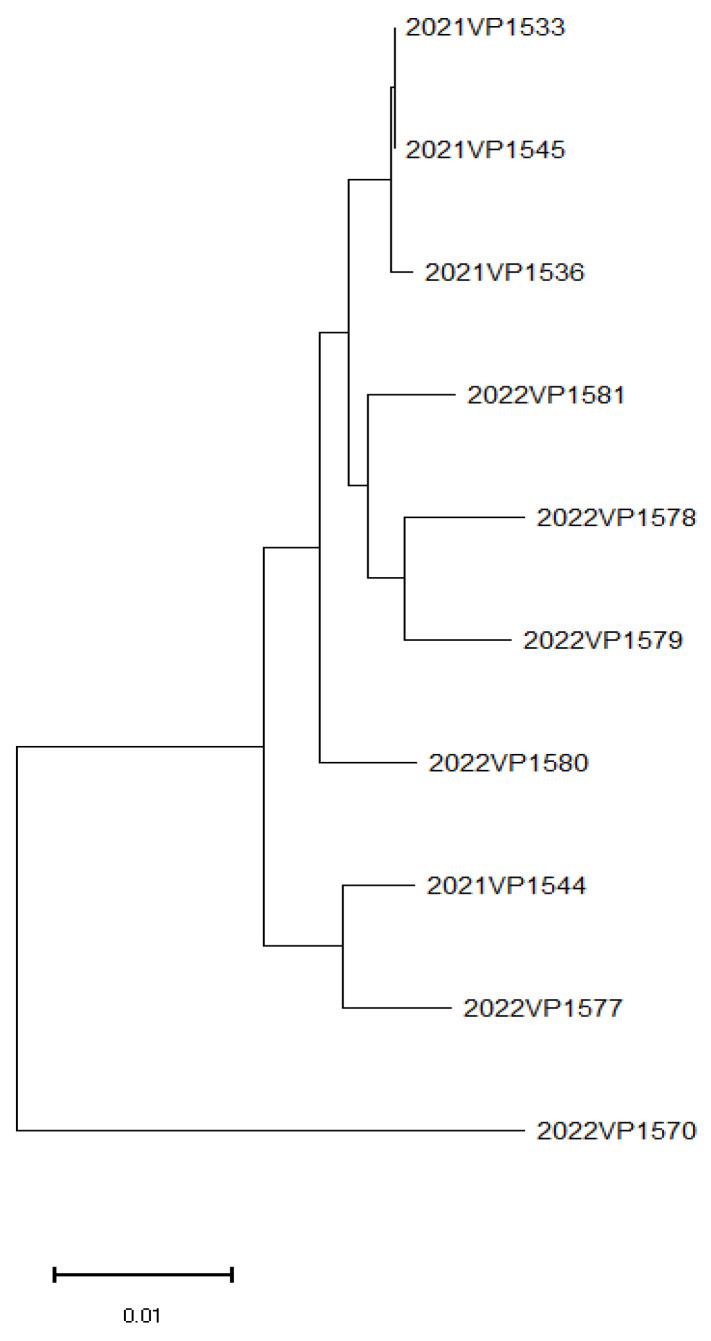
Phylogenetic tree of *Vibrio parahaemolyticus* isolates based on WGS.

Phylogenetic analysis based on WGS showed clustering of isolates based on their MLSTs. Notably, isolates 2021VP1533 and 2021VP1545 were closely related, whereas 2022VP1570 displayed the greatest genetic divergence among the analyzed strains. The phylogenetic tree generated using the MEGA X software is presented in Figure 1.

### 3.5. Virulence Factor Analysis

Virulence factors were analyzed using WGS data and the VFDB. A total of 256 unique virulence genes were screened, among which 177 genes were detected (119 genes consistently in all isolates and 58 genes in only some isolates). The remaining 79 genes were either not detected in any samples or lacked defined names for related genes despite being listed in VFclass and the VFDB. These unidentified genes were not presented separately. Genes that were absent in all isolates were excluded from the analysis. The results are presented in Table 8 and Table 9.

#### 3.5.1. Commonly Identified Virulence Factors

Virulence factors consistently detected in all isolates included *tlh*, a key virulence factor in *V. parahaemolyticus* associated with hemolytic activity and cytotoxicity. Additionally, *T3SS1* genes, which are involved in bacterial invasion and cytotoxicity, were detected in all strains. These genes were identified in all isolates, regardless of their geographic or temporal origin. The presence of these common virulence factors was further verified by PCR, indicating that these genes are stably and consistently maintained in all isolates (Table 8).

Among the 58 commonly detected genes shared across all WGS-analyzed isolates, the following categories were identified: adherence (mannose-sensitive hemagglutinin [MSHA type IV pilus]-related genes: *mshA*, *mshE*, *mshF*, etc.), antiphagocytosis (capsular polysaccharide-related genes: *cpsA*, *cpsB*, etc.), chemotaxis and motility (flagella-related genes: *cheA*, *cheB*, *flaA*, etc.), iron uptake (enterobactin receptor-related genes: *irgA*, *vctA*, etc.; heme receptors-related genes: *hutA*, *hutR*, etc.; periplasmic binding protein-dependent ABC transport systems-related genes: *vctC*, *vctG*, etc.), quorum sensing (cholera autoinducer-1-related genes: *cqsA*), secretion system (EPS type II secretion system-related genes: *epsC*, *epsE*, etc.; T3SS1 secreted effectors-related genes: *vopQ*, *vopR*, etc.; T3SS1-related genes: *sycN*, *tyeA*, etc.), and toxin (thermolabile hemolysin-related gene: *tlh*).

#### 3.5.2. Individually Identified Virulence Factors

Several virulence genes were detected only in specific isolates. Notably, T3SS1 effectors exhibited variability among strains, whereas *tdh* and *T3SS2* genes were absent in all isolates (Table 9). In addition, considerable genetic diversity was observed among strain-specific virulence factors, indicating that certain isolates possessed distinct sets of virulence-related genes.

The strain-specific virulence factors were categorized as follows. Adherence-related genes included those associated with mannose-sensitive hemagglutinin (MSHA type IV pilus), such as *mshC* and *mshD*, as well as type IV pilus-related genes like *pilA*, and genes associated with the tad locus (*Haemophilus*), such as *tadA*. Antiphagocytosis-related genes comprised capsular polysaccharide-associated genes, including *cpsC*, *rmlA*, *rmlB*, *rmlC*, *wbfT*, *wbfU*, *wbfV*/*wcvB*, *wbfY*, *wbjD*/*wecB*, *wecA*, *wecC*, *wza*, *wzb*, and *wzc*. Chemotaxis and motility-related genes involved flagella-associated genes such as *flaD*, *flaG*, *flgA*, *flgB*, *flgC*, *flgM*, *flgN*, *fliJ*, and *fliQ*.

Iron uptake-related genes included those involved in periplasmic binding protein-dependent ABC transport systems, such as *vctD*. Quorum sensing-related genes included the autoinducer-2-associated gene *luxS*. Secretion system-related genes were subdivided into several groups: those associated with the EPS type II secretion system, including *epsI* and *epsM*; T3SS1-associated genes, such as *vcrH*, vcrR, *virG*, *vscB*, *vscF*, *vscS*, *vscX*, and *vscY*; VAS effector proteins-related genes, including *hcp-2*, *vgrG-2*, and *vgrG-3*; and genes related to the VAS type VI secretion system, such as *vasA*, *vasB*, *vasD*, *vasE*, *vasG*, *vasH*, *vasJ*, and *vasK*. In addition, genes associated with TTSS (SPI-1 encode) included *invF*.

Toxin-related genes included those associated with phytotoxin phaseolotoxin, such as *cysC1*. Colonization and immune evasion-related genes included capsule biosynthesis and transport-associated genes like *kpsF*. Endotoxin-related genes included lipooligosaccharide-associated genes, such as *lgtF*, while immune evasion-related genes included lipopolysaccharide-associated genes, such as *acpXL*. Other genes included those associated with O-antigen, such as *fcl*, *manB*, and *cpsB*.

## 4. Discussion

This study investigated the prevalence, antimicrobial resistance, and virulence characteristics of *V. parahaemolyticus* isolated from sashimi fillet, offering insights into seafood safety risks [23,24]. Despite the relatively low overall isolation rate (3.4%), the detection of *V. parahaemolyticus* in widely consumed seafood, such as flatfish and rockfish sashimi, underscores the need for enhanced monitoring and regulatory measures to prevent potential foodborne illnesses [25,26].

While sashimi is often considered a high-risk food, the actual infection risk depends on multiple factors, including bacterial load, host susceptibility, and handling and storage practices [27]. Given that *V. parahaemolyticus* infections range from mild gastroenteritis to life-threatening septicemia, even a low contamination rate necessitates ongoing surveillance and risk management [23].

In Korea, foodborne illnesses associated with *V. parahaemolyticus* are frequently reported, particularly during the summer months when seafood consumption increases [28]. Previous studies have demonstrated that elevated seawater temperatures enhance the expression of virulence genes such as *tdh* and *trh*, potentially increasing the pathogenic potential of *V. parahaemolyticus* [29]. National surveillance data indicate a seasonal surge in *V. parahaemolyticus*-induced gastroenteritis in South Korea, accounting for approximately 7% of foodborne outbreaks between 2002 and 2017, with 80.8% of cases occurring between July and September [28]. The relatively low isolation rate observed in this study does not negate the risk, as even small bacterial loads can lead to outbreaks when favorable conditions, such as improper storage or temperature abuse, are present [25]. Moreover, asymptomatic carriers and subclinical infections may contribute to underreporting of cases, necessitating further epidemiological studies.

The findings of this study are consistent with previous research indicating the widespread presence of *V. parahaemolyticus* in seafood [30]; however, certain differences were noted. Its prevalence in flatfish and rockfish sashimi underscores the potential risk of exposure through raw seafood consumption in Korea. Compared with studies from East and Southeast Asia, where higher prevalence rates of *V. parahaemolyticus* have been reported in raw seafood [31,32], regional differences in the prevalence of *tdh* and *trh* genes have been reported, with higher detection rates in Southeast Asia compared with those in Korea [32]. These variations may reflect differences in seafood handling practices and environmental conditions. The lower detection rate in this study may be attributed to differences in seafood handling practices, distribution chains, and storage conditions. Further comparative research is needed to evaluate the impact of these factors on bacterial prevalence in different regions [33].

Among the 17 isolates, 58.8% exhibited resistance to ampicillin, consistent with previous reports from various countries [34]. Ampicillin resistance in *V. parahaemolyticus* was primarily attributed to the production of β-lactamase enzymes, which enables the degradation of β-lactam antibiotics [35]. The ampicillin resistance observed is primarily attributed to the production of TEM-type β-lactamase, which is often plasmid-encoded, suggesting the potential for horizontal gene transfer among marine bacterial populations. Given that β-lactams are among the most commonly used antibiotics in both human medicine and aquaculture, their widespread use likely contributes to the persistence of resistant strains in marine environments [36]. The presence of resistance genes suggests the potential for horizontal gene transfer, which could facilitate the spread of AMR among marine bacterial populations [37].

Interestingly, despite the widespread use of oxytetracycline in aquaculture [38], no tetracycline-resistant strains were identified in this study. This contrasts with previous studies reporting tetracycline resistance rates of 6–17% in China, Italy, and other regions [32,39]. The absence of tetracycline resistance may be attributed to differences in antibiotic usage policies, regional variations in aquaculture practices, or the presence of competing microbial communities that influence resistance gene acquisition [40,41]. Future studies should assess antibiotic residue levels in aquaculture environments to better understand the selective pressures driving resistance development.

The genetic diversity observed in MLST analysis highlights the evolutionary dynamics of *V. parahaemolyticus* in seafood-related environments [10,42,43]. The presence of multiple STs suggests that *V. parahaemolyticus* isolates originate from diverse phylogenetic backgrounds, potentially influenced by environmental factors, seafood trade routes, and regional variations in bacterial populations [43].

Notably, the identification of ST114 in two isolates suggests the possibility of localized dissemination within the seafood supply chain. Given that these isolates were obtained from seafood purchased on the same day in Gyeongsang Province (Busan), this may indicate a shared contamination source at the wholesale or processing level. Furthermore, these two isolates exhibited identical PCR results, reinforcing the likelihood that they originated from the same contamination source. This genetic and molecular similarity suggests a strong epidemiological link, emphasizing the need for further tracking of seafood distribution networks to determine whether ST114 represents a dominant strain in the region or a sporadic occurrence.

In addition, the emergence of novel or previously unreported STs underscores the ongoing genomic evolution of *V. parahaemolyticus*. This may be driven by environmental changes, selective pressures in marine ecosystems, or bacterial adaptation to different hosts [44,45]. The potential implications of these novel STs in terms of virulence, antimicrobial resistance, and ecological fitness warrant further genomic and functional studies.

Environmental factors, particularly rising ocean temperatures, may further contribute to the increasing prevalence and persistence of *V. parahaemolyticus* in seafood [29]. Warmer waters and altered marine ecosystems could exert selective pressures that facilitate genetic adaptation, potentially driving the emergence of novel STs observed in this study [46]. Previous studies have demonstrated that elevated temperatures can induce mutations in key virulence genes, altering bacterial pathogenicity. Given the projected impacts of climate change on marine ecosystems, integrating climate data into seafood safety monitoring programs could improve early warning systems for *Vibrio* outbreaks [47].

From a food safety perspective, these findings highlight the necessity for enhanced microbial surveillance and antibiotic resistance monitoring in seafood [48]. Routine WGS-based surveillance could provide real-time insights into the genetic evolution of *V. parahaemolyticus* strains, enabling early detection of emerging threats. Additionally, stricter regulations on antibiotic use in aquaculture should be enforced to minimize the selection pressure for resistant strains. Consumer education on proper seafood handling and storage is equally crucial in reducing foodborne risks.

To further mitigate the risks associated with *V. parahaemolyticus* contamination in sashimi, routine WGS-based surveillance should be implemented [49]. Additionally, stricter hygiene regulations in seafood processing facilities, such as Hazard Analysis and Critical Control Points (HACCPs) systems, should be enforced to minimize contamination risks [50]. Consumer education on proper handling and storage of raw seafood is equally essential in reducing public health risks [51].

## 5. Conclusions

This study investigated the prevalence, virulence genes, and antimicrobial resistance of *V. parahaemolyticus* in sashimi fillets, emphasizing the public health risks associated with raw seafood consumption. Despite a low isolation rate, the detection of virulence genes (*tlh*, *trh*) and ampicillin resistance highlights the necessity of continuous surveillance and targeted intervention strategies to mitigate the risk of foodborne illness.

As seafood consumption continues to increase, systematic microbiological surveillance, stringent antibiotic regulations in aquaculture, and enhanced food safety education are imperative for reducing *V. parahaemolyticus*-related risks. WGS-based surveillance offers a robust approach for detecting emerging antimicrobial resistance patterns and virulence trends, thereby facilitating timely and evidence-based public health interventions.

Future research should extend beyond sashimi to include other high-risk seafood products, such as shellfish and crustaceans, to provide a more comprehensive assessment of *V. parahaemolyticus* prevalence and resistance dynamics. Furthermore, given the growing evidence that rising ocean temperatures influence bacterial persistence and transmission, investigations into the impact of climate change on the distribution and adaptation of *V. parahaemolyticus* are warranted. Strengthening regional and international collaboration in seafood safety research is essential to developing effective strategies for controlling the global dissemination of antimicrobial-resistant pathogens.

## Figures and Tables

**Table 1:** Regional distribution of seafood samples collected in 2021–2022.

	Region				
Year	Jeonla	Gangwon	Chungcheong	Seoul–Gyeonggi	Gyeongsang
2021	40	6	63	71	70
2022	40	10	50	80	70
Total	80	16	113	151	140

**Table 2:** Antimicrobial agents, tested concentration ranges, and breakpoints.

Antimicrobial Subclass	Antimicrobial Agent (Abbreviation)	Range Tested	Breakpoint
Aminoglycosides	Gentamicin (GEN)	1–64	≥16 ^(1)^
	Streptomycin (STR)	16–128	ND *
Aminopenicillin	Ampicillin (AMP)	2–64	≥32 ^(1)^
β-lactam/β-lactamase inhibitor combinations	Amoxicillin/clavulanic acid (AMC)	2/1–32/16	≥32/16 ^(1)^
Cephamycin	Cefoxitin (FOX)	1–32	≥32 ^(1)^
Cephalosporin III	Cefotaxime (CTX)	0.5–8	≥4 ^(1)^
	Ceftazidime (CAZ)	1–16	≥16 ^(1)^
Cephalosporin IV	Cefepime (FEP)	0.25–16	≥16 ^(1)^
Carbapenem	Meropenem (MEM)	0.25–4	≥4 ^(1)^
Fluoroquinolone	Ciprofloxacin (CIP)	0.12–16	≥4 ^(1)^
Folate pathway inhibitors	Trimethoprim/Sulfamethoxazole (SXT)	0.12/2.38–4/76	≥4/76 ^(1)^
	Sulfisoxazole (FIS)	16–256	≥512 ^(1)^
Phenicols	Chloramphenicol (CHL)	2–64	≥32 ^(1)^
Polymyxins	Colistin (COL)	2–16	ND
Quinolone	Nalidixic acid (NAL)	2–128	ND
Tetracyclines	Tetracycline (TET)	2–128	≥16 ^(1)^

* ND: not determined. ^(1)^ according to CLSI guidelines [15].

**Table 3:** Details of primers used in this study.

Target Gene	Primer	Sequence (5′→3′)	Target Amplicon Size (bp)	Reference
*tlh*	tl-F	AAAGCGGATTATGCAGAAGCACTG	405	[16]
	tl-R	GCTACTTTCTAGCATTTTCTCTGC		
*tdh*	tdh-F	GTAAAGGTCTCTGACTTTTGGAC	259	[16]
	tdh-R	TGGAATAGAACCTTCATCTTCACC		
*trh*	trh-F	TTGGCTTCGATATTTTCAGTATCT	500	[16]
	rth-R	CATAACAAACATATGCCCATTTCCG		
*T3SS1*	vscN1-F	GGGGCTGTGGTGCCGGGCGTA	1325	[17]
	vscN1-R	GGGGCGATGCCTTTCAGTTGAGC		
*T3SS2α*	vscN2-F	AAACGTACTCACCGACTCGAATG	1120	[17]
	vscN2-R	TGAAATCGTTAAGGTGACAGGC		
*T3SS2β*	vcrD2-F	GGTAACACTGCCTGGTGTGGTCATCG	1594	[19]
	vcrD2-R	GTCTCTCAAAGTCTTCAAACTCACCTGC		
*T6SS1*	icmF1-F	AGTACCGCCTGCCAATAAGACAAC	411	[20]
	icmF1-R	GACGCATCGGCAAACTCAACAG		
T6SS2	icmF2-F	AATGGATTGGGACTAGGGAGGTTG	452	[20]
	icmF2-R	TACGCGTTATTTGCTGCTTGAGA		

**Table 4:** *Vibrio parahaemolyticus* isolates from domestic fish samples (2021–2022).

Year	Origin	Category	No. of Samples	No. of Isolates (%)
2021	Domestic	Fish	250	8(3.2)
2022	Domestic	Fish	250	9(3.6)
Total			500	3.4%

**Table 5:** Antimicrobial resistance assay results of *V. parahaemolyticus* isolates.

Antimicrobial Subclass	Antimicrobial Agent (Abbreviation)	No. of Isolates (%)
		Resistant
Aminoglycosides	Gentamicin (GEN)	0 (0)
	Streptomycin (STR)	ND *
Aminopenicillin	Ampicillin (AMP)	10 (58.8)
β-lactam/β-lactamase inhibitor combinations	Amoxicillin/clavulanic acid (AMC)	0 (0)
Cephamycin	Cefoxitin (FOX)	0 (0)
Cephalosporin III	Cefotaxime (CTX)	0 (0)
	Ceftazidime (CAZ)	0 (0)
Cephalosporin IV	Cefepime (FEP)	0 (0)
Carbapenem	Meropenem (MEM)	0 (0)
Fluoroquinolone	Ciprofloxacin (CIP)	0 (0)
Folate pathway inhibitors	Trimethoprim/Sulfamethoxazole (SXT)	0 (0)
	Sulfisoxazole (FIS)	0 (0)
Phenicols	Chloramphenicol (CHL)	0 (0)
Polymyxins	Colistin (COL)	ND *
Quinolone	Nalidixic acid (NAL)	ND *
Tetracyclines	Tetracycline (TET)	0 (0)

* ND: not determined.

**Table 6:** Virulence genes and secretion systems in *Vibrio parahaemolyticus* isolates detected by polymerase chain reaction.

Sample	*tlh*	*tdh*	*trh*	T3SS1	T3SS2α	T3SS2β	T6SS1	T6SS2
21_VP_1530	+	−	+	+	−	−	+	+
21_VP_1531	+	−	+	+	−	−	−	+
21_VP_1533	+	−	+	+	−	−	+	+
21_VP_1536	+	−	+	+	−	−	+	+
21_VP_1542	+	−	+	+	−	−	−	+
21_VP_1544	+	−	+	+	−	−	−	+
21_VP_1545	+	−	+	+	−	−	+	+
21_VP_1774	+	−	+	+	−	−	+	+
22_VP_1570	+	−	+	+	−	−	−	+
22_VP_1572	+	−	+	+	−	−	+	+
22_VP_1574	+	−	+	+	−	−	−	+
22_VP_1576	+	−	−	+	−	−	−	+
22_VP_1577	+	−	+	+	−	−	+	+
22_VP_1578	+	−	−	+	−	−	−	+
22_VP_1579	+	−	−	+	−	−	+	+
22_VP_1580	+	−	−	+	−	−	+	+
22_VP_1581	+	−	−	+	−	−	−	+
Total	17/17(100)	0/17(0)	12/17(70.6)	17/17(100)	0/17(0)	0/17(0)	9/17(52.9)	17/17(100)

+, positive (detected); −, negative (not detected).

**Table 7:** ResFinder and MLST analysis using WGS data for antimicrobial resistance and genetic typing.

Sample ID	Genetic Background	Antimicrobial	Class	WGS-Predicted Phenotype	ST	Nearest STs
21_VP_1533	blaCARB-40	AMX, AMP, PIP	Beta-lactam	Resistant	114	
21_VP_1536	blaCARB-21	AMX, AMP, PIP	Beta-lactam	Resistant	Unknown	2902, 1989, 114, 2170
21_VP_1544	blaCARB-26	AMX, AMP, PIP	Beta-lactam	Resistant	2447	
21_VP_1545	blaCARB-21	AMX, AMP, PIP	Beta-lactam	Resistant	114	
22_VP_1570	blaCARB-48	AMX, AMP, PIP	Beta-lactam	Resistant	Unknown	3085, 281
22_VP_1577	blaCARB-45	AMX, AMP, PIP	Beta-lactam	Resistant	917	
22_VP_1578	blaCARB-46	AMX, AMP, PIP	Beta-lactam	Resistant	1256	
22_VP_1579	blaCARB-33	AMX, AMP, PIP	Beta-lactam	Resistant	Unknown	992, 396, 3223
22_VP_1580	blaCARB-33	AMX, AMP, PIP	Beta-lactam	Resistant	1823	
22_VP_1581	blaCARB-33	AMX, AMP, PIP	Beta-lactam	Resistant	Unknown	2590, 2621, 2125, 1956, 358

**Table 8:** Virulence factors commonly identified in V. parahaemolyticus through WGS-based VFDB analysis.

VF Class	Virulence Factor	Related Genes
Adherence	Mannose-sensitive hemagglutinin (MSHA type IV pilus)	*mshA*, *mshE*, *mshF*, *mph*, *mshH*, *mshI*, *mshJ*, *mshK*, *mshL*, *mshM*, *mshN*
	Type IV pilus	*pilB*, *pilC*, *pilD*
Antiphagocytosis	Capsular polysaccharide	*cpsA*, *cpsB*, *cpsD*, *cpsE*, *cpsF*, *cpsG*, *cpsH*, *cpsI*, *cpsJ*, *wbfV/wcvB*
Chemotaxis and motility	Flagella	*cheA*, *cheB*, *cheR*, *cheV*, *cheW*, *cheY*, *cheZ*, *filM*, *flaA*, *flaB*, *flaI*, *flgD*, *flgE*, *flgF*, *flgG*, *flgH*, *flgI*, *flgJ*, *flgK*, *flgL*, *flhA*, *flhB*, *flhF*, *flhG*, *fliA*, *fliD*, *fliE*, *fliF*, *fliG*, *fliH*, *fliI*, *fliK*, *fliL*, *fliN*, *fliO*, *fliP*, *fliR*, *fliS*, *flrA*, *flrB*, *flrC*, *motA*, *motB*, *motX*, *motY*
Iron uptake	Enterobactin receptors	*irgA*, *vctA*
	Heme receptors	*hutA*, *hutR*
	Periplasmic binding protein-dependent ABC transport systems	*vctC*, *vctG*, *vctP*
Quorum sensing	Cholerae autoinducer-1	*cqsA*
Secretion system	EPS type II secretion system	*epsC*, *epsE*, *epsF*, *epsG*, *epsH*, *epsJ*, *epsK*, *epsL*, *epsM*, *epsN*, *gspD*
	T3SS1 secreted effectors	*vopQ*, *vopR*, *vopS*
	T3SS1	*sycN*, *tyeA*, *vcrD*, *vcrG*, *vcrV*, *virF*, *vopB*, *vopD*, *vopN*, *vscA*, *vscC*, *vscD*, *vscG*, *vscH*, *vscI*, *vscJ*, *vscK*, *vscL*, *vscN*, *vscO*, *vscP*, *vscQ*, *vscR*, *vscT*, *vscU*, *vxsC*
Toxin	Thermolabile hemolysin	*tlh*

**Table 9:** Detailed analysis of non-common VFDB genes across isolates.

VF Class	Virulence Factor	Related Genes	2021				2022					
			VP1533	VP1536	VP1544	VP1545	VP1570	VP1577	VP1578	VP1579	VP1580	VP1581
Adherence	Mannose-sensitive hemagglutinin (MSHA type IV pilus)	*mshC*										
		*mshD*										
	Type IV pilus	*pilA*										
	Tight adherence locus (*Haemophilus*)	*tadA*										
Antiphagocytosis	Capsular polysaccharide	*cpsC*										
		*rmlA*										
		*rmlB*										
		*rmlC*										
		*wbfT*										
		*wbfU*										
		*wbfV/wcvB*										
		*wbfY*										
		*wbjD/wecB*										
		*wecA*										
		*wecC*										
		*wza*										
		*wzb*										
		*wzc*										
Chemotaxis and motility	Flagella	*flaD*										
		*flaG*										
		*flgA*										
		*flgB*										
		*flgC*										
		*flgM*										
		*flgN*										
		*fliJ*										
		*fliQ*										
Iron uptake	Periplasmic binding protein-dependent ABC transport systems	*vctD*										
Quorum sensing	Autoinducer-2	*luxS*										
Secretion system	EPS type II secretion system	*epsI*										
		*epsM*										
	T3SS1	*vcrH*										
		*vcrR*										
		*virG*										
		*vscB*										
		*vscF*										
		*vscS*										
		*vscX*										
		*vscY*										
	VAS effector proteins	*hcp-2*										
		*vgrG-2*										
		*vgrG-3*										
	VAS type VI secretion system	*vasA*										
		*vasB*										
		*vasD*										
		*vasE*										
		*vasG*										
		*vasH*										
		*vasJ*										
		*vasK*										
	TTSS (SPI-1 encode)	*invF*										
Toxin	Phytotoxin phaseolotoxin	*cysC1*										
Colonization and Immune evasion	Capsule biosynthesis and transport	*kpsF*										
Endotoxin	Lipooligosaccharide	*lgtF*										
Immune evasion	Lipopolysaccharide	*acpXL*										
Others	O-antigen	*fcl*										
		*manB*										
		*cpsB*										

Gray shading indicates detected genes; blank cells indicate genes not detected. Empty VF class and Virulence Factor cells indicate the same group as above.

## Data Availability

The genome assemblies of *Vibrio parahaemolyticus* isolates analyzed in this study have been deposited in the NCBI database under BioProject accession number PRJNA1254427, with associated BioSample accession numbers SAMN48128082 to SAMN48128091. The data will be made publicly available upon publication.
